# Wearable Devices and Explainable Unsupervised Learning for COVID-19 Detection and Monitoring

**DOI:** 10.3390/diagnostics13193071

**Published:** 2023-09-28

**Authors:** Ahmad Hasasneh, Haytham Hijazi, Manar Abu Talib, Yaman Afadar, Ali Bou Nassif, Qassim Nasir

**Affiliations:** 1 Department of Natural, Engineering, and Technology Sciences, Faculty of Graduate Studies, Arab American University, Ramallah P-600-699, Palestine; ahmad.hasasneh@aaup.edu; 2 Department of Informatics Engineering, CISUC-Centre for Informatics and Systems of the University of Coimbra, University of Coimbra, 3030-790 Coimbra, Portugal; 3 Intelligent Systems Department, Palestine Ahliya University, Bethlehem P-150-199, Palestine; 4 College of Computing and Informatics, University of Sharjah, Sharjah 27272, United Arab Emirates; mtalib@sharjah.ac.ae (M.A.T.); yafadar@sharjah.ac.ae (Y.A.); anassif@sharjah.ac.ae (A.B.N.); nasir@sharjah.ac.ae (Q.N.)

**Keywords:** AI, COVID-19 detection, clustering, unsupervised learning, wearables

## Abstract

Despite the declining COVID-19 cases, global healthcare systems still face significant challenges due to ongoing infections, especially among fully vaccinated individuals, including adolescents and young adults (AYA). To tackle this issue, cost-effective alternatives utilizing technologies like Artificial Intelligence (AI) and wearable devices have emerged for disease screening, diagnosis, and monitoring. However, many AI solutions in this context heavily rely on supervised learning techniques, which pose challenges such as human labeling reliability and time-consuming data annotation. In this study, we propose an innovative unsupervised framework that leverages smartwatch data to detect and monitor COVID-19 infections. We utilize longitudinal data, including heart rate (HR), heart rate variability (HRV), and physical activity measured via step count, collected through the continuous monitoring of volunteers. Our goal is to offer effective and affordable solutions for COVID-19 detection and monitoring. Our unsupervised framework employs interpretable clusters of normal and abnormal measures, facilitating disease progression detection. Additionally, we enhance result interpretation by leveraging the language model Davinci GPT-3 to gain deeper insights into the underlying data patterns and relationships. Our results demonstrate the effectiveness of unsupervised learning, achieving a Silhouette score of 0.55. Furthermore, validation using supervised learning techniques yields high accuracy (0.884 ± 0.005), precision (0.80 ± 0.112), and recall (0.817 ± 0.037). These promising findings indicate the potential of unsupervised techniques for identifying inflammatory markers, contributing to the development of efficient and reliable COVID-19 detection and monitoring methods. Our study shows the capabilities of AI and wearables, reflecting the pursuit of low-cost, accessible solutions for addressing health challenges related to inflammatory diseases, thereby opening new avenues for scalable and widely applicable health monitoring solutions.

## 1. Introduction

The evolution of the Severe Acute Respiratory Syndrome Coronavirus 2 (SARS-CoV-2) outbreak has drastically affected everybody’s life around the globe, even after the decline of cases. As of the end of September 2023, over 770,778,396 confirmed cases and over 6,958,499 deaths have been reported globally [1]. The emergence of various viral variants, such as Delta, B.1.640, and Omicron (with subvariants like XBB.1.5), has further complicated the situation. COVID-19 can have a wide range of severities, from asymptomatic to fatal illness, and can affect multiple organ systems [2]. The Coronavirus Disease 2019 (COVID-19) affects individuals’ physical health, and with the ability to cause changes in the cardiovascular and respiratory systems, it could lead to multiple organ failures [3]. The severity of COVID-19 can be an asymptomatic, subclinical, mild, moderate, severe, or fatal illness [4].

Given the severity of the virus, the high spread, and the emerging variants, the COVID-19 pandemic has developed a significant challenge for global healthcare systems, causing some to collapse. Islam et al. [5] argued that even with the high vaccination rates in some countries, hospitals and healthcare centers are still overcrowded and perceived as virus carriers. Therefore, to respond efficiently to this outbreak while maintaining the essential health services running, mandatory requirements must be fulfilled such as (a) the complete isolation of infected individuals and the continuous monitoring of the severe cases, and special (b) attention and monitoring to older people (>60 years) and those who are vulnerable to different diseases, and notably even adolescent and young adults.

The detection and monitoring of COVID-19 is a critical aspect in the control of the disease and the minimization of the infection rate. The prioritization of hospitalization is also facilitated via effective detection and monitoring strategies. One of the most widely adopted techniques for the classical detection of COVID-19 is the reverse transcription polymerase chain reaction (RT-PCR) test. However, in certain countries or healthcare systems that are overwhelmed, the viability of RT-PCR testing may be limited [6]. Despite its accuracy and sensitivity, the RT-PCR test may present challenges due to its prolonged turnaround time and the requirement for trained personnel to perform the nasal swab with reliability [7].

In recent years, biosensors have seen significant growth, particularly in the integration of wearable devices such as smartwatches and bracelets. These wearables offer the capability for the continuous measurement and monitoring of physiological data, including heart rate, skin temperature, respiratory rate, and oxygen saturation, as well as physical activity data such as step count. The physiological data have demonstrated the ability to reflect various changes induced by the infections and diseases in individuals. For instance, Heart Rate Variability (HRV), also referred to as Pulse Rate Variability (PRV) in photoplethysmogram (PPG) technologies, has been shown to serve as a potential marker of viral infections, including COVID-19 [8]. Recent studies, like the one reported in [9], have indicated that the elevated heart rate measurements obtained from smartwatches can be utilized in epidemiological studies to track the spread of respiratory illnesses. For instance, Heart rate (HR), as measured via wrist-worn wearables using optical sensors like PPG, has the potential to be a valuable cross-disciplinary biomarker with practical clinical applications in psychology, psychiatry, and medicine [9].

Millions of people worldwide use affordable wearable devices, mainly smartwatches [10], to track their physiological and physical activities, including heart rate, sleeping periods, and stress [11]. However, the physiological data are sensitive to many factors, such as environmental, motion, or psychological ones (e.g., stress, distraction, and many others) [12]. Therefore, integrating multimodal feature space could mitigate this effect, especially when using Machine Learning (ML) techniques that could recognize various patterns associated with specific events (e.g., infection). Therefore, it is relevant to investigate the role of these devices in detecting and monitoring the progress of the infection in a reasonable time as potential support for the classical approaches of detection and monitoring.

Various studies (e.g., [13,14,15]) have used AI and ML techniques to predict or monitor COVID-19 using the physiological and physical activity wearable data while integrating volunteers’ self-reporting into the feature space. Although these studies performed reasonably, most relied on supervised learning techniques. Yakimovich et al. [16] showed that data labeling in supervised learning techniques is costly and challenging, particularly in biomedical applications (which need highly reliable labeling). Annotating the clinical data could be subject to bias, inaccuracy, and non-transparency. Therefore, unsupervised learning could be a potential solution to avoid such challenges and unveil the data patterns and characteristics we have not thought of before more reliably.

Avoiding biases in data labeling with supervised learning does not imply unsupervised learning is fully unbiased and transparent. Explaining clustering model results is essential in critical applications, such as clinical models, like the one we introduce in this paper. Kelly et al. [17] argue that even if we understand the mathematical notions and basics of the AI models, it is still a challenge to interrogate “how” and “why” the model made a certain decision. In such a way, experts can recognize inappropriate reasoning of the model, unacceptable results, and potential bias, and try to mitigate that.

This paper introduces a new explainable unsupervised learning framework that aims to detect COVID-19 infection in individuals (young adults). The framework uses interpretable clustering techniques based on individuals’ physiological (HR & HRV) data and physical activity acquired from wearable smartwatches. We focused on young adults (18–24) in this study for many reasons: (a) the lack of studies that focus on this group, (b) many studies (e.g., [18,19]) showed that the risk factor of younger adults infected with COVID-19 to progress severely could be high, and (c) the youth group is dynamic in mobility and interaction, leading to a higher probability of contagiousness. Therefore, detecting COVID-19 in young adults is relevant and essential.

The explainability in this model comes in two flavors: an abstract level explanation using auto-generated rules from a decision tree supervised learning model [20] and a personal-level explanation. The abstract level does not provide a narrative explanation for non-expert users or patients but mathematical inequality signs. On the contrary, the user-level explanation makes use of the GPT (Generative Pre-training Transformer) Davinci model, which generates a human-like text to interpret the results of clustering in simple wordings. In a recent article [21], the author states that, with caution, Natural Language Processing (NLP) chatbots have the potential to revolutionize the field of medical writing by automating the process. Another recent study [22] shows that such NLP models can describe the situation but not the underlying knowledge. Therefore, in our framework, we limited the use of Davinci GPT-3 to describe the existing generated rules from the first layer of the model explanation. The methodology in this paper explains that use in further detail.

The contributions of this paper are as follows:

- This paper (to our knowledge) is the first to compare unsupervised learning methods in detecting and monitoring COVID-19 using wearable devices while automatically interpreting the results using two different approaches of explainability, including the use of Davinci GPT-3.
- This paper focuses on an essential population, young adults, to investigate the role of AI and wearable devices in controlling the spread of the disease, especially in asymptomatic patients, via detection and monitoring.
- The paper provides an internal and external evaluation of the clustering techniques, including using “supervised learning” state-of-the-art classifiers.

Thus, in contrast to the other published works in this domain, our research represents one of the early initiatives to show the potential of unsupervised learning, extending beyond mere anomaly detection, to uncover concealed patterns at various stages of COVID-19 illness. Although recent studies (e.g., [15]) have underscored the application of unsupervised machine learning for the early detection and diagnosis of COVID-19, extracting valuable insights directly from the illness, they have not explored this approach within the realm of wearable data in cost-effective settings, as shown in our work.

The rest of the paper is organized as follows: (a) Current Approaches, where we discuss the state of the art in detecting and monitoring COVID-19 via various electronic means and AI; (b) Methods and Dataset, which provides information about volunteers, data acquisition, and pre-processing, feature engineering, and clustering algorithms; (c) Results and Discussion, which shows the results of this study while providing implications in the context of COVID-19 detection and monitoring; (d) Threats to Validity, which discusses the internal, external, and construct threats, and (e) Conclusion and Future directions.

## 2. Current Approaches

Current technology-based approaches for COVID-19 detection, diagnosis, and monitoring primarily utilize either sensor-based techniques or image-based techniques. The latter approach, utilizing X-ray and Computed Tomography (CT-scan) images, has demonstrated higher accuracy in detecting the disease and monitoring patient symptoms, as reported in [20,23,24]. In recent times, wearable devices have emerged as a new sensor-based technology for the early detection of COVID-19 and the remote monitoring of infected patients. These devices offer real-time measurements, such as heart rate (HR), respiratory rate (RR), temperature, and physical activity, with minimal involvement of healthcare professionals and users. The pre-processed data from these devices can serve as input features for training artificial intelligence (AI) and machine learning (ML) models for the early detection and diagnosis of COVID-19, as demonstrated by Hijazi et al. [25].

Several machine-learning techniques have been applied to the sensor data for COVID-19 early detection and disease control. For instance, Hoang et al. [11] used body temperature monitoring and human activity recognition during quarantine to control the spread of the virus and monitor patient conditions. Changes in RR have been found to play a critical role in detecting and monitoring the progression of COVID-19, as demonstrated in studies [12,13]. These studies also showed the effectiveness of jointly using RR with the other wearable features, such as cough [14] or HR, SpO2, and temperature [7].

While many studies have used supervised ML methods for COVID-19 analysis, diagnosis, and monitoring, very few have utilized unsupervised techniques, as reported in [15,16,18]. Boussen et al. [15] proposed an unsupervised machine learning model, based on the Gaussian mixture model (GMM), for COVID-19 triage and monitoring. The breathing frequency and saturation signals were found to serve as an alert source at least 48 h prior to intubation for infected patients, with an accuracy of 87.8% for intubation recognition. Another study [16] developed an unsupervised health monitoring system for predicting and analyzing the health status of COVID-19 patients, utilizing a K-means clustering algorithm. However, this system requires the widespread production of specialized microcontrollers, limiting its applicability. In contrast, our work uses low-cost and widely available smartwatches (e.g., Fitbit), with the potential challenge of precision and sensitivity mitigated via filtering methods, ML techniques, and representative feature space.

Unsupervised learning techniques have also been applied to the COVID-19-related data, such as the prediction of the COVID-19 pandemic spread around the world using GMM [18]. Another example is the use of the K-means clustering algorithm for COVID-19 countries’ cluster analysis [19]. However, a large body of supervised ML techniques has been proposed for the remote monitoring of COVID-19 patients’ health statuses and conditions, such as the use of wearable Internet of Things (IoT) sensors, cloud, and web layers [26], or the use of deep learning models for monitoring the progression of COVID-19 [27,28,29]. Despite these advancements, further research is necessary to fully understand and address the complex nature of COVID-19 and its impact on public health.

Current methods for detecting and diagnosing COVID-19 still suffer from several issues. The classical screening and diagnosing of COVID-19 can be a time-consuming process and costly [30,31]. In particular, a systematic review of diagnosis methods for COVID-19 has shown that traditional diagnostic approaches like Reverse Transcription Polymerase Chain Reaction (RT-PCR) tests can be time-consuming and resource-intensive, which leads to delays in results and increased costs [30]. Another recent study highlights the main challenges in detecting and diagnosing COVID-19 using traditional approaches. This study shows that these classical methods typically involve complex data collection, analysis, and processing, which could contribute to the overall cost and time required to perform the diagnosis process [31]. On the other hand, a supervised machine learning method can potentially induce unreliable results in COVID-19 detection and diagnosis [32] due to mislabeling of or incomplete data, which forces the model to learn incorrect patterns. Furthermore, supervised models may not generalize well to new or evolving variants of the virus, which may reduce their effectiveness in real-world scenarios.

In contrast, biosensors and wearable technology, including smartwatches, have shown promise in advancing and enhancing the detection and diagnosis of inflammatory diseases like COVID-19 [33,34,35]. Specifically, the authors in [33] have shown the potential of wearable sensing technology in monitoring the long-term effects of COVID-19, which often involve inflammatory issues. Another research work explores the application of wearable biosensors in detecting various body signals, including those related to inflammatory responses, which can be crucial in diagnosing diseases like COVID-19 [34]. In addition to that, recent work in [35] highlights the overall advancement of wearable sensor technology, which can be adapted for detecting and diagnosing inflammatory diseases, including COVID-19. Consequently, using biosensors and wearable technology will contribute to improving the detection and diagnosis of COVID-19 by providing continuous monitoring and valuable data to healthcare professionals.

Despite the large arsenal of work that contributed to the early detection of COVID-19, detecting and monitoring patients infected with COVID-19 remains a significant challenge. Although several studies showed reasonable performance using supervised ML techniques applied to the wearable physiological data, there is a clear lack of studies that used explainable unsupervised learning in such applications. This confirms that investigating unsupervised machine learning methods, such as the K-means clustering algorithm, followed by an interpretable method that explains the results, is relevant and worthy of further research attempts. In light of this, this paper proposes using clustering algorithms utilizing wearable data to detect and monitor COVID-19 cases with two layers of result explanations (i.e., abstract and user level), which we believe is the first study to investigate in that direction. The following sections provide a detailed view of the data, the methods, and the evaluation measures.

## 3. Dataset and Methods

This section provides an overview of the dataset used followed by an explanation of the methodology.

### 3.1. Dataset Description

A research group led by the University of Sharjah, in cooperation with the University of Coimbra (Portugal), Arab American University, and Palestine Ahliya University, conducted a longitudinal study among 28 individuals (still recruiting more volunteers) who were asked to wear a Fitbit-Versa 2 smartwatch to collect the following data: (a) Heart Rate (HR), (b) Physical activity (e.g., step count), and (c) Sleeping data. The overall goal was to analyze their physiological and physical activity data via Artificial Intelligence methods to detect and monitor COVID-19 infection, possibly before the symptom’s onset. For this purpose, the research group adhered to the University of Sharjah’s ethical committee’s rigid requirements concerning non-intrusive data acquisition from humans, which were fulfilled. The first step was to contact volunteers via emails, personal networks, and social network posts. Then, we received volunteers’ consent stating that the collected physiological and physical activity data would be solely used for research targets and would never be shared or sold to any third party for business purposes.

The volunteers downloaded the Athena CX platform on which our application (WeDetect) was published and disseminated. The volunteers were then asked to synchronize their Fitbit smartwatch with the Athena CX platform. WeDetect has two types of subjective questionnaires: (a) a first-time questionnaire to collect demographic data, vaccination, and disease history (if it exists), and (b) a time-triggered survey in which questions about symptoms and self-assessment were present. Moreover, the wear time of the wearable and the response time to the questions was also recorded. Notably, the COVID-19 PCR or Antigen rapid test results were also acquired as a binary value (positive/negative) with their corresponding dates.

### 3.2. Volunteers

The study involved 29 recruited volunteers, out of which 11 were found to be infected with COVID-19. The dataset used in the study comprised a total of 106,847 readings, including heart rate (HR), steps, and sleep data. Specifically, there were 50,596 readings from the COVID-19 infection period and 56,251 readings from the healthy period of the volunteers. This balanced dataset facilitated the formation of more homogeneous clusters of the data.

The objective of this research was to investigate the capability of AI algorithms in detecting and monitoring COVID-19 infections among young adults. To achieve this, the study recruited volunteers from the University of Sharjah (UoS) in the United Arab Emirates, consisting of university students and young workers. Throughout the study period, the volunteers were provided with Versa 2 devices. The age of the volunteers averaged 21.3 ± 1.91, with both males and females represented in the sample at 54.5% and 45.5%, respectively.

### 3.3. Data Preprocessing and Feature Engineering

**Figure 1:**
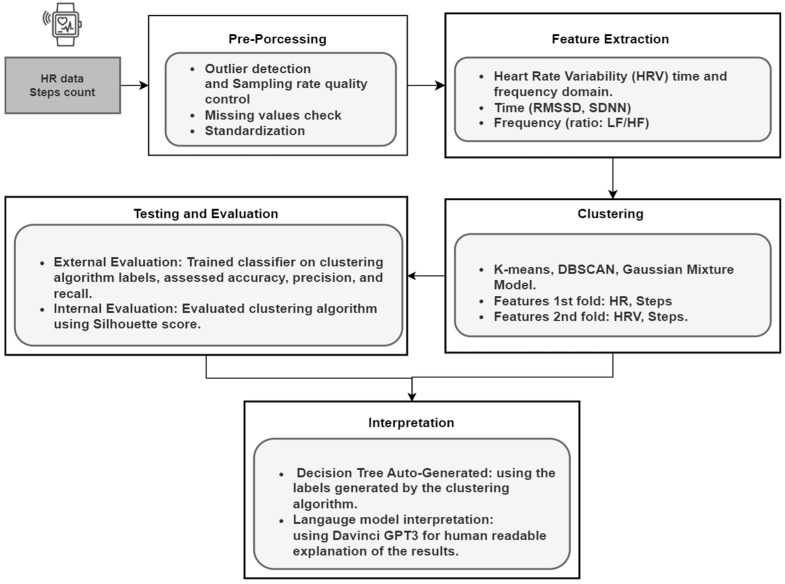
Schematic Representation of the proposed framework.

Figure 1 illustrates the sequential steps involved in preprocessing, clustering, and interpreting the results of the HR data analysis. Given that the HR data collected from wearables may not be as accurate as those obtained from dedicated cardiac sensors, we conducted thorough preprocessing to ensure data quality. HRV or more precisely PRV is computed via a sequence of steps.

In the context of heart rate variability (HRV), specifically pulse rate variability (PRV), a series of computational steps are employed.

Initially, the RR intervals, measured in seconds, are calculated using the formula 60 divided by the heart rate (HR) [36]. Once these RR intervals are obtained, a subsequent process involves subtracting the duration of every (n − 1)th RR interval from the duration of the nth RR interval. This computation yields approximate RR intervals.

Subsequently, a root-mean-square calculation is applied to these intervals over a 5 min time window. This procedure provides an estimation of RMSSD (root mean square of successive differences), which is closely aligned with the RMSSD values accessible via the Fitbit Web API. This approach offers valuable insights into heart rate variability.

To analyze the spectral frequency components, the Fast Fourier Transform (FFT) is employed. Frequency bands are automatically delineated, including the high-frequency (HF, 0.15–0.4 Hz) and low-frequency (LF, 0.04–0.15 Hz) bands. The FFT process incorporates a Hanning window is applied to the derived RR interval data by utilizing a 5 min duration, followed by smoothing spline interpolation. This interpolation serves to address potential gaps or irregularities in the data, enhancing the robustness of the analysis [37].

Second, we visually inspected the data to identify any irregularities or artifacts. Subsequently, the following steps were performed.

#### 3.3.1. Outlier Detection

Commercial wearables often capture motion or environmental artifacts [9], which can distort HR readings. To address this issue, we excluded any HR values that fell outside the acceptable range (heart rate > 200 or heart rate < 30) [38]. Then, further outliers are detected using Z-scores, denoted as *Z_i*, calculated as the standard deviation away from the mean, denoted as μ. A predefined threshold, denoted as τ, is set to identify the extreme values. The data points with absolute Z-scores greater than τ are considered outliers [39].

#### 3.3.2. Missing Data Detection

Missing values in the HR data can lead to the loss of important individual characteristics, such as peak values or patterns. To mitigate this, we employed the HeartImp method [39] to impute any missing values. Furthermore, considering that Fitbit Versa 2 records the HR data at a sampling rate of 1 Hz, we aggregated the readings to achieve a minute-by-minute resolution, rather than at the second level. This adjustment was made to optimize the computational cost of the modeling process without losing the signal power. Additionally, to account for the variations in the data scales between HR and the other measurements, such as step count, we applied data standardization. This step aimed to normalize the data and eliminate any potential biases introduced by differing scales [23], as shown in Formula (1).

(1) $$x*=\frac{x-\mu }{\sigma }$$

where μ and σ are the data samples’ mean and standard deviation, respectively.

By implementing these preprocessing techniques, we aimed to enhance the accuracy and reliability of the HR data analysis, ensuring a more robust foundation for subsequent modeling and interpretation.

Concerning the Heart Rate Variability extraction, and according to the established literature in explaining the used features in clinical use [7], we extracted specific time-domain and frequency-domain features described in Table 1 below.

After extracting the HR and deriving the HRV from it, the data is investigated in two folds: (a) HR and Step Count, and (b) HRV features’ interaction with HR. For the first fold, it is well established that elevated HRs are witnessed during illness and can be detected using wearable devices [13]. Thus, we wanted to examine the ability of clustering techniques (e.g., k-means) to separate abnormal readings (i.e., higher HR with lower step count) from regular readings (i.e., higher HR with higher step count or lower HR with lower step count). However, since HR is sensitive to physical activity, we selected the Resting Heart Rate (RHR) by assuming 2:00 am to 6:00 am is the typical resting period [41]. Therefore, the first fold represents RHR by keeping an eye on the step count for successive minutes (e.g., 10–20) and the day/nighttime of the measurement to ensure the readings are RHR. Bogu et al. [14] detected abnormal RHR during COVID-19 in 92% of the patients using wearable devices and deep learning methods, showing that abnormal RHR appeared 5 days before the symptoms’ onset.

However, since HR and RHR are static markers, we wanted to examine a dynamic marker from the HR, which is the Heart Rate Variability (HRV). Thus, regarding the second fold of analysis, we examine the ability of clustering algorithms to recognize relevant changes in the HRV features (time and frequency domain) associated with COVID-19 infection, as described above in Table 1.

### 3.4. Clustering Algorithms

This work investigates the ability of unsupervised techniques, namely Clustering, to unveil the data patterns associated with normal and abnormal health statuses relative to COVID-19 infection in cases where labeling and ground-truth reliability are existing challenges. Clustering divides the data points into a specific number of groups (i.e., clusters) based on similar properties. In our words, we are examining the matching behavior of RHR with the physical activity and the HRV time and frequency domain to detect and monitor the abnormal cases relative to COVID-19 infection. However, since the RHR and HRV changes are nonspecific to COVID-19 infection or inflammatory response, it was required to gather volunteers’ responses to subjective assessment questionnaires to augment the decision of clustering algorithms. To interpret the results of different clustering algorithms, we used unsupervised Decision Tree-generated rules [20], as will be described later.

Table 2 describes the clustering algorithms we examined and justifies the reasons for choosing them for the comparison in our framework while showing the essential parameters of each of them.

The idea is to investigate the best clustering performance that fulfills the following criteria: (a) accurate separation of the data points, (b) good performance, and (c) tolerance to data variability, as in the case of the physiological data. However, evaluating the clustering performance against these criteria is not straightforward. We used the Silhouette score, a familiar method proposed by Rousseeuw et al. [45], to assess the data separability and, thus, the clustering model, without using external ground truth or labels. The concept of this score is to estimate the difference between the tightness of the within-cluster and separation from the other clusters. Let us take a sample *s(i)* for *i* ∈ *I*; the silhouette score is calculated as:

(2) $$s\left(i\right)=\frac{b\left(i\right)-a(i)}{max(a\left(i\right),\;b(i))}$$

where *a*(*i*) is the average distance between *i* and all the other data points of the cluster where i ∈ cluster, and *b*(*i*) is the minimum of the average distances between *i* and all the other data points in each cluster. Value 1 means that the data point or the sample is correctly assigned to the cluster and away from the other neighboring clusters. The best silhouette score is 1, and the worst is −1.

In our evaluation, we used the silhouette score as a preliminary criterion to decide the best clustering algorithm that fits our data.

### 3.5. Model Explanation

We employed two types of explainability in our model. The abstract level explainability depends on training a decision tree classifier utilizing the resulting clusters from the k-means (i.e., the best-performing algorithm). The following figure illustrates the process of assigning the resulting clusters of the K-means, training a decision tree classifier, and deriving the rules.

The resulting interpretation is in the form of mathematical inequality signs, which has the following limitations: (a) although it gives a first layer of reasoning about a given cluster, it does not prioritize interpretability during cluster creation; (b) at the normal user-level, mathematical notions would not be a favorable form of interpretation; and (c) the decision tree’s discretization of continuing values, which may result in losing useful information for the interpretability. Therefore, we added a second layer of model explanation using the Natural Language Understanding Model, namely the GPT-3 Davinci model.

Listing 1 provides a pseudo-code that outlines the clustering rules’ generation process. This process combines unsupervised machine learning (K-means clustering algorithm) to discover clusters and supervised machine learning (Decision Tree) to extract the rules that describe the relationship between the input variables and the cluster labels, providing insight into the different stages of COVID-19 illness.

**Listing 1.** Clustering Rules Generation.

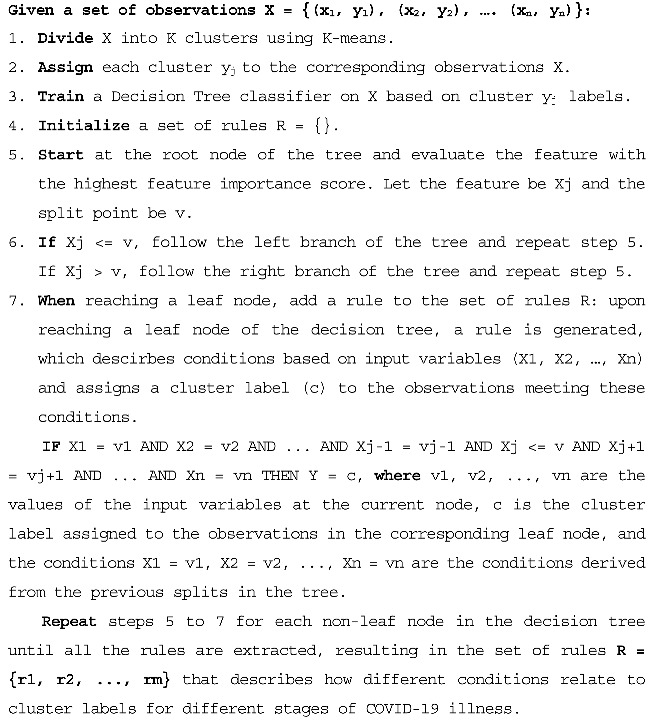



To utilize the OpenAI GPT Davinci model API, we used the openai library and set the API key as shown in Listing 2 below.

**Listing 2.** Importing OpenAI and Setting API Key.

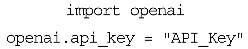



We then used the openai.Completion.create function, as shown in Listing 3, to specify the engine to be used, which in this case was the “text-davinci-002” engine, given that we fed the prompt with well-established context from the medical resources as appears below.

The GPT model operates based on the principles established via the decision tree algorithm and endeavors to correlate the numerical values of HR and the steps (i.e., high or low) with the medical data it has been trained on, so as to arrive at conclusions regarding the probability of infection. To verify the validity and precision of the responses generated by the model, we solicited the opinion of medical experts to evaluate the accuracy of the output against established medical knowledge and standards. It is important to note that each instance of the model results in a unique explanation, generated via the process of synthesis. Given the ongoing nature of research into language models and their application, it must be emphasized that the results generated by this model should not be relied upon as the sole basis for making medical decisions. Rather, these results should be regarded as supplementary information that may assist in reaching informed conclusions. The findings from the decision tree-derived rules, the GPT-3 model, and the medical expert evaluations will be presented in the subsequent section.

**Listing 3.** Generating Explanation Using OpenAI API.

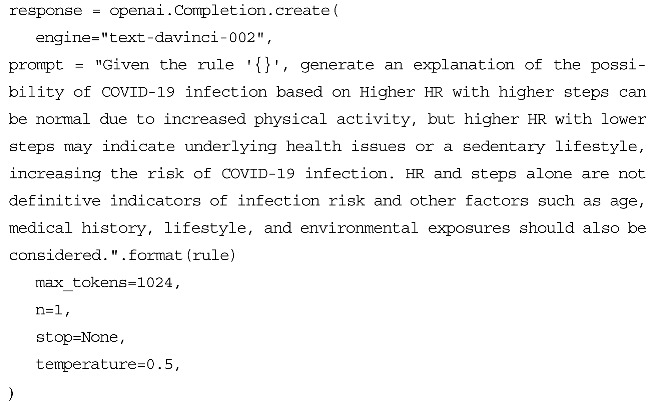



In summary, we introduced two types of explainability as follows.

(a) Enhancing Model Explainability:

Initially, we employed the outcomes of the k-means clustering technique to train a decision tree classifier. This procedure involved assigning the resulting clusters from the k-means method, training a decision tree classifier, and formulating rules expressed via mathematical inequalities. However, this approach had its limitations, as it did not prioritize ease of understanding during cluster formation and relied on math-heavy notations that might be challenging for non-experts.

(b) Bringing Clarity with Natural Language:

To make our model’s results more understandable and user-friendly, we introduced an additional layer of interpretation using OpenAI’s GPT-3 Davinci model. This language model follows the same principles as the decision tree algorithm but conveys explanations in natural language, making them more accessible and intuitive for users.

## 4. Results and Discussion

The evaluation results of this work consider the two folds of data clustering shown in Figure 1, which are (1) HR and Physical Activity and (2) HRV with HR. The three folds’ evaluation results will be presented in the following structure:

(a) **Local analysis results:** at this level, we show the results of each clustering algorithm as follows:
  - K-means evaluation starts with the k estimation using the elbow method, visualizes the clustering results and centroids, and estimates the silhouette score. Moreover, we introduce the decision tree-generated rules to interpret the clustering results.
  - DBSCAN evaluation starts with the epsilon values’ effect, visualizes the clustering results and the noise detected, and estimates the silhouette score. Likewise, we provide the interpretation of the results.
  - GMM evaluation, starting with visualizing the clustering results and providing an interpretation of different distributions’ Clustering.
(b) **Global analysis:** at this level, we conduct a comparison between the models based on internal evaluation using their silhouette score and external evaluation using accuracy, precision, and recall, after using the Clustering-generated labels to train a set of classifiers.
(c) **Explainability evaluation:** since the explanation is what reaches the user at the end, it is essential to validate it.

**Figure 2:**
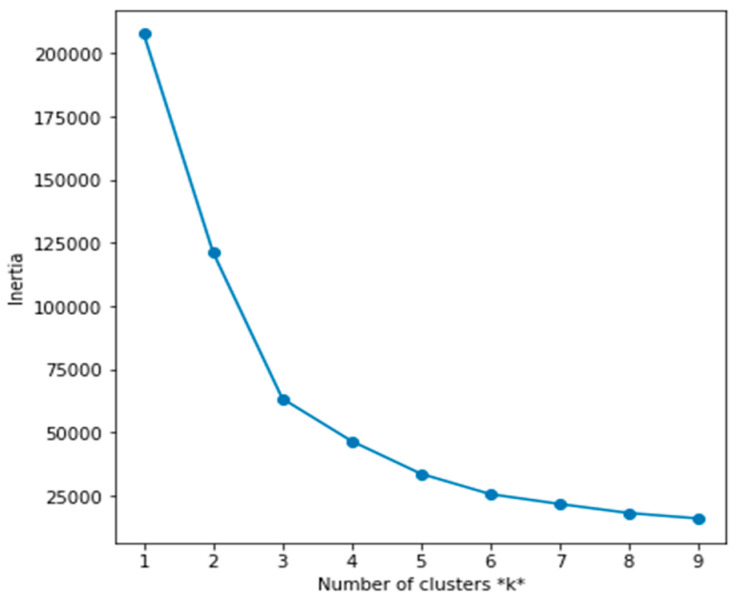
Elbow method to select the best k.

Starting with the k-means algorithm, Figure 2 shows the best number of clusters (i.e., k), which lies at 3. Therefore, we started with three clusters in k-means and visualized the results of the k-means and the other clustering algorithms mentioned above. We just specified the number of components of the GMM to be 3 for a better comparison between the clustering algorithms.

**Figure 3:**
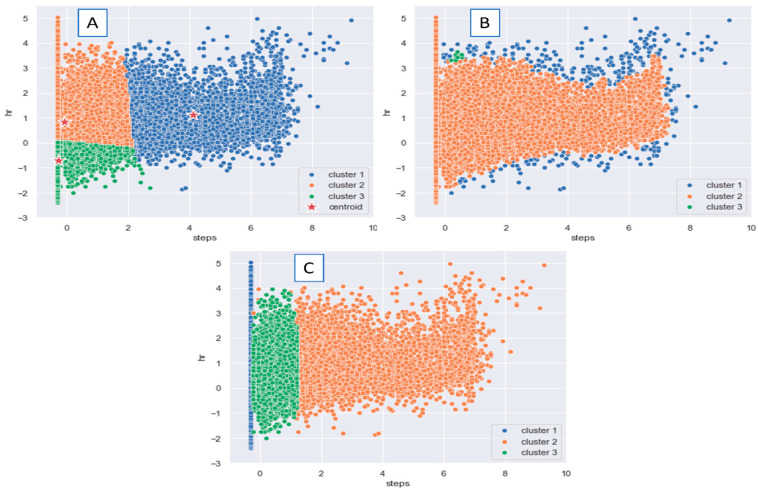
HR and Steps Count applied to (**A**): K-means k = 3, (**B**) DBSCAN eps = 0.2, and (**C**) GMM, no. of components = 3.

Figure 3 depicts the visualization results of the three chosen clustering algorithms: (A) k-means for the first fold of data RHR with steps, (B) DBSCAN with the same data and with eps = 0.2, and (C) GMM where the number of components equals 3. As we can see in the rightmost part, in the k-means case, the first cluster (i.e., the blue) exhibits higher steps with higher HR values. This is a typical case wherein physical movement induces complementary interaction between the influences of the vagal inhibitory and the increase in sympathetic excitatory influences, and thus, higher RHR values [46,47]. Cluster 3 in k-means appears in the green region and represents relatively low physical activity (i.e., steps) and reduced RHR.

This case was further checked in terms of the time of measurements (to ensure it has minimal physical activity). The analysis suggests that these readings represent the RHR in the regular case (i.e., the healthy status). The region of interest in this work is found in the second cluster (orange) of the k-means. We witness relatively higher RHR values with lower physical activity in this cluster. Mayer et al. [48] hypothesized in their work on the role of consumer wearables in detecting physiological changes that an increase in the basal HR (or RHR) is a result of increases in RHR while individuals are in febrile states. However, since HR and even the RHR are sensitive to the other physiological or environmental factors, this result should be integrated with other contextual information, such as the questionnaire responses, to make a final decision.

In (B) DBSCAN and (C) GMM, we could not find interesting patterns of clusters as we could in the k-means. Visual inspection provided initial insights into the potential clusters and groupings within the data, suggesting that k-means clustering could be a suitable approach for further exploration. While visual inspection alone is not a definitive scientific method, it serves as an important exploratory step in our research process, guiding our choice of the k-means algorithm as an initial approach to uncover underlying patterns in the data.

To obtain a macroscopic view of the different clustering techniques, an internal evaluation of the clustering algorithms was performed from the Silhouette Score perspective.

**Figure 4:**
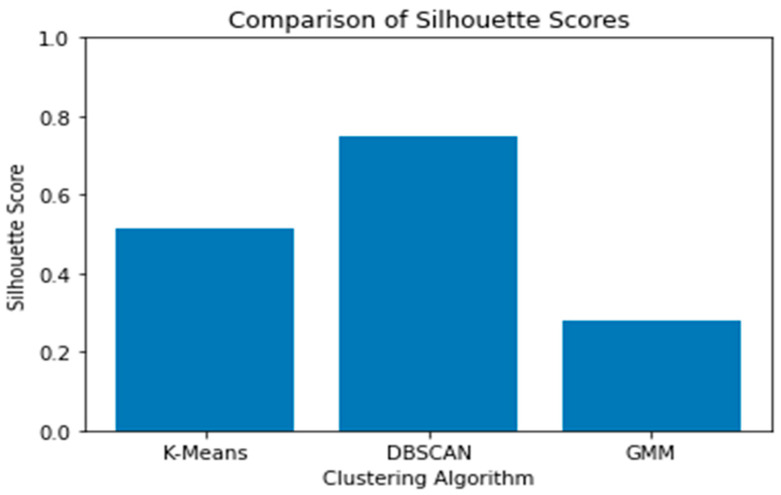
Silhouette Score Comparison.

Silhouette represents the goodness of Clustering by exhibiting how similar a data point is to its cluster (i.e., cohesion). Figure 4 shows the comparison between the chosen three algorithms.

As we notice in Figure 4, both K-means and DBSCAN perform well. However, it is wise to invest more analysis attempts with the k-means, especially because we could understand the behavior of the data points and relate the results to human-readable medical explanations. However, before applying the external evaluation of the K-means, we want to understand the clustering algorithm behavior. We used decision trees to extract the K-means’ rules used to separate the data points. The decision tree takes the cluster labels produced by the K-means, trains a decision tree (chosen for its explainability) on these labels, and then creates the rules for better explainability. Table 3 shows three distinct rules of the decision tree with a pruning level of 0.05.

As shown in Table 3, Rule 1 represents the blue cluster in Figure 3A, which refers to the increasing RHR and physical activity levels. Rule 2, which refers to the “orange cluster,” exhibits the abnormal situation (potentially COVID-19 infected), which shows the increase in the HR above a threshold of 85.5 with reduced activity steps <= 35.5. The table shows that 99% of the data points that satisfy Rule 2 are in cluster 1 (or the orange cluster). Finally, the last cluster (the green) shows the normal case of minimal physical activity associated with reduced RHR.

**Figure 5:**
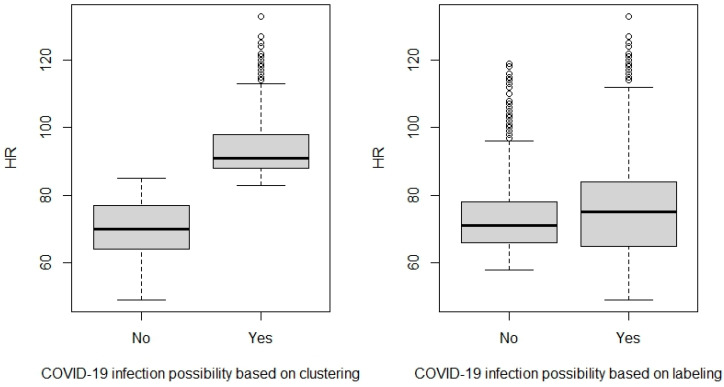
HR Distribution Using Clustering Labels and Human Labels.

However, since the thresholds here do not exactly express the medical thresholds of the abnormalities, we took the clustering labels of the K-means algorithm and transformed them into binary labels. The cluster label with increased RHR and a decreased step count is considered abnormal, but otherwise is normal. In Figure 5, we visualize the distribution of RHR relative to the normal and abnormal clustering labels. We conduct the same for the manual labels that were produced by our team based on the dates of the COVID-19 positive and negative results (i.e., label 1 for the COVID-19 positive test period and 0 after the first negative test). We notice from Figure 5 that in the clustering labels, the RHR in the COVID-19 period and the healthy period shows significant statistical differences with a *p*-value of 0.00001 using the Wilcoxon signed rank test after testing the normality using the Shapiro–Wilk test (which exhibited non-normality).

On the other hand, we tested the RHR in the COVID-19 infection and non-infection periods as our team labeled according to the PCR-reported test dates. Both labels (the Clustering and the human) show significant differences between the two groups, but with more discrimination using the clustering label. The Wilcoxon test indicates a significant statistical difference between the two groups with a *p*-value of 0.0028.

We manually inspected the dataset that includes the volunteers’ subjective health self-assessment for more insights to compare the two labels (Clustering and human-produced). We found that most human labels are based on the negative and positive COVID-19 tests, considering the period with fatigue, headache, stress, and cough after the negative test as “0”, representing the inaccurate behavior of humans in supervised learning methods. In contrast, the clustering labels could detect 77–80% of these cases by labeling them as “1”, which means the abnormal or unhealthy status. This result would lead us to “carefully” consider that clustering algorithms would be a promising potential to detect and monitor COVID-19 infection with minimal reliance on humans in labeling.

### 4.1. External Evaluation Using Supervised Learning

For a more robust evaluation of the K-means, chosen among the other two algorithms for extensive evaluation, in Table 4, we show the results of examining the clustering labels by training a set of supervised classifiers. We used leave-one-out cross-validation to avoid any bias caused by the inter- and intra-variability of the heart rate signals.

As we notice from the table above, the labels generated from K-means could achieve good results as an external evaluation of the clustering algorithm, especially in SVM.

For a more reliable result, we applied the same features and labels to a random classifier which shuffles the labels using permutation test scores. This method generates a null distribution by calculating the accuracy of the classifier on 1000 permutations of the dataset, where the features remain fixed while the labels undergo different permutations. After performing the permutation test, a *p*-value of 0.0091 represents the percentage of permutations for which the score (i.e., the accuracy) is greater than the accuracy obtained using the original data (without permutations).

### 4.2. HRV Clustering Evaluation

We transformed the HR data into HRV to extract time and frequency domain features. The HR data points were converted to the RR interval data by multiplying the inverse of the HR with 60. We applied a low-pass filter for the RR interval data to remove the noise. Using a 30 s sliding time window, we could extract the most promising features associated with inflammatory processes, as established in the literature [49,50,51]. One of these features is the standard deviation of mean RR intervals (SDNN). Hasty et al. [51] showed that the drop of the SDNN value of more than 40% is followed by more than a tripling of the C-Reactive Protein (CRP), which is used as a tracker for the inflammatory process of COVID-19. CRP is associated with disease progression and thus could be used as a vital indicator in monitoring or detecting the disease.

**Figure 6:**
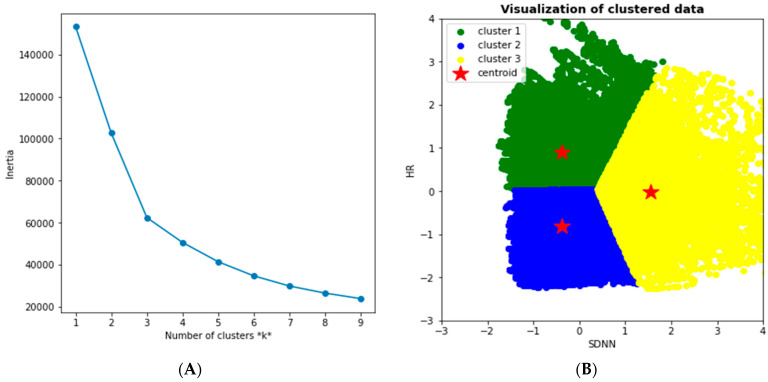
(**A**) Elbow Method for the SDNN of the HRV and HR (second fold of data), and (**B**) resulting Clustering for SDNN and HR.

Since the K-means showed promising results in the first fold of data (HR and Step count), we used them again with the HRV and HR data. The Elbow method provided us with the best k for the SDNN; the HR data was three, as shown in Figure 6A. We visualized the resulting Clustering of SDNN and the HR data, as shown in Figure 6B. In this Clustering, we notice that the reduced SDNN and increased HR (i.e., the green cluster) correspond to the normal status where the body has little parasympathetic activity due to potential physical activities (high HR). The blue cluster, however, corresponds to an abnormal status translated into lower SDNN while HR is at rest. In contrast, the yellow cluster represents another segment of a potentially healthy status. The higher the SDNN (or HRV in general), the more resilient the bodies of young adults adapt to inflammatory changes [52].

**Figure 7:**
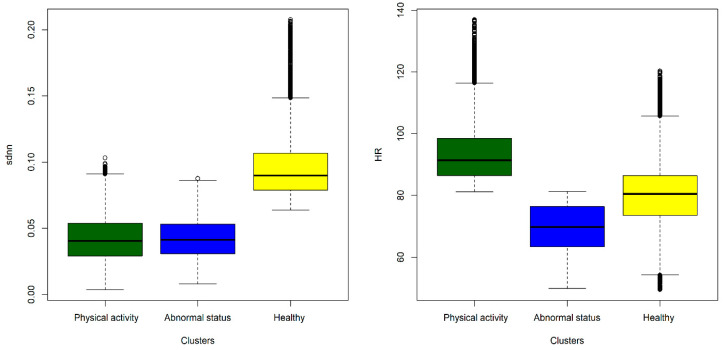
SDNN and HR Distribution Among Clusters.

In Figure 7, we analyzed the distribution of SDNN and HR among the resulting clusters of the K-means. We observed that the HR tends to increase while the SDNN decreases in the case of increased physical activity.

In contrast, in the abnormal status (i.e., potentially COVID-19), we notice a decrease in the SDNN while HR is at rest. Finally, HR and SDNN are at their expected values in the healthy cluster. In other words, there is no observed increase in the HR and a decrease in HRV (i.e., SDNN).

The silhouette score of the SDNN and HR clustering resulted in 0.39, which is relatively fair.

In the second fold of data (HR and HRV), we followed the same analogy in interpreting the first fold of data clustering (i.e., HR and physical activity), which labels a decision tree using the resulting clusters and generates the rules. Table 5 shows these rules, which could provide us with the following interpretations: a) in class 0 or rule 1, the status can be referred to as physical activities, where HR is increasing, and SDNN (i.e., HRV) is decreasing. The second cluster or rule represents the potential abnormal status via the reduced SDNN while the HR is at rest. The final cluster or the final rule exhibits the likely healthy behavior dominated by low HR and increased HRV (i.e., SDNN).

These rules may not provide precise thresholds due to the limited data set. However, expanding the dataset should be helpful from an application point of view. A more conservative argument about the results and the framework is discussed in the next section, highlighting the threats to Validity.

### 4.3. Language Model Interpretation Evaluation

To evaluate the quality of responses generated via the GPT-3 models in the context of cardiovascular diseases, we developed a comprehensive questionnaire. This questionnaire was carefully designed to assess the accuracy and alignment of the model-generated replies with medical facts and common sense.

To ensure a rigorous evaluation, we sought the expertise of six medical professionals specialized in cardiovascular diseases. Each expert independently evaluated the responses on a scale ranging from 1 to 5. A rating of 1 indicated an inaccurate response, while a rating of 5 represented the highest level of accuracy.

The findings from the expert evaluations revealed that the GPT-3 models achieved a modest accuracy rate of only 36% in representing a second layer of model interpretation. This indicates that there is room for improvement in terms of the model’s ability to provide reliable explanations for cardiovascular disease-related queries.

To address this limitation, it is crucial to dedicate additional efforts toward refining the model. This could involve a combination of enhanced engineering practices and potentially conducting fine-tuning specific to cardiovascular diseases. By incorporating these measures, we aim to enhance the reliability and accuracy of the model’s explanations, ultimately providing valuable insights and guidance in the field of cardiovascular disease.

## 5. Threats to Validity

Although this study introduced a new way to look at the data from an unsupervised learning angle to avoid human inaccuracies, the study has limitations that we know and handle seriously.

**Construct Validity:** The use of wearable devices’ sensors in acquiring the HR data might introduce validity threats due to the use of a non-direct method to measure heart signals. Instead, we utilize wearables utilizing photoplethysmography (PPG) technology to estimate blood volume changes as a surrogate for Electrocardiography (ECG). Various studies (e.g., [53]) have demonstrated the feasibility of using PPG to extract an estimation of HR from smartwatches and bracelets. Additionally, it is important to note that our study relied on the HR values provided by the Fitbit device’s internal algorithm rather than direct access to the raw PPG signal, introducing the possibility of limited construct validity in representing HRV. However, well-established studies like those reported in [54,55] show that the PPG-derived features obtained from both the wearables, including Fitbit, and the research devices showed a strong and statistically significant positive correlation, along with the observed acceptable heart rate accuracy (<±10%) among the devices.

Moreover, HR and HRV are sensitive to many factors, such as stress, physical activity, and other environmental factors. We tried to overcome this by gathering daily subjective assessments of the volunteers’ health. This step has helped us validate the Clustering or the manual labeling.

Another aspect of the construct’s Validity is the clustering algorithms, namely the K-means, which we adopted on its excellent performance. Although K-means is an easy algorithm to implement and run, it has several shortcomings, such as (a) the initial seeds have a substantial impact on the results and performance, (b) the data dimensionality has a substantial impact on the results, and (c) rescaling the data changes the results considerably. We tried to avoid these situations by running the algorithms 30 times to see the best iteration (i.e., seed) in terms of the least square error. Moreover, we addressed the data dimensionality by investigating two features simultaneously.

**Internal Validity:** in our study, we could not control the environment in which our volunteers live, and thus, we could not have a full view of their behavior when wearing the smartwatches (whether they wear them regularly). To mitigate this, we added a feature in our platform to indicate the wear time of our volunteers. Whenever a long break in leaving the wearable was noticed, we notified the volunteer by email to wear it again.

**External Validity:** the limited number of volunteers is considered a challenge in this study. The researchers are aware of the number of volunteers’ challenges; therefore, they are still recruiting more volunteers to validate the results further. However, this had one advantage: we investigated the COVID-19 detection and monitoring of young adults, which was not sufficiently explored in the literature. It narrowed the scope of the study to young adults and could not extrapolate the results to older people, who are the most vulnerable sector in our communities.

## 6. Conclusions and Future Work

COVID-19 monitoring and detection using wearable devices (e.g., smart watches) have been a growing trend in recent studies in the field. Most of these studies used Machine Learning and AI to augment the detection or prediction decision space. However, the majority focused on supervised learning, which could be less efficient and reliable due to potential human errors in labeling and time consumption in doing the process.

In this study, we introduced a new framework to investigate the role of unsupervised learning in recognizing the patterns of normal and abnormal behaviors of HR and HRV measures in the context of COVID-19 infection. We recruited 29 volunteers, of which 11 were infected with COVID-19 during the study. We provided them with a Fitbit Versa 2 wearable to be worn during the study to acquire the HR, steps count, and sleep data via an application. The application gathered a subjective assessment of their health daily. After extracting the data, we examined three known clustering algorithms: K-means, DBSCAN, and the Gaussian Mixture Model (GMM). The first data fold was the HR and the physical activity indexed by the step count. The second data fold was HR and HRV represented by SDNN.

We used supervised machine learning as an external evaluation of the K-means by training these classifiers on the resulting cluster labels. The results showed that K-means has the best performance in terms of Silhouette score (0.55) and interpretability relative to known medical facts from established papers. SVM has 0.884 ± 0.005, 0.80 ± 0.112, and 0.817 ± 0.037 representing the best accuracy, precision, and recall, respectively. We performed permutation score classification to ensure the results were not achieved by chance. After shuffling the labels, the resulting *p*-value was 0.0099; this means we can reject the null hypothesis that there are no actual dependencies between features and labels. Finally, we used the decision tree classifier to generate the rules of the K-means cluster labels to interpret the results. In comparison to the other studies within this field, our research is among the early explorations that demonstrate the potential of unsupervised learning in uncovering hidden patterns associated with various stages of COVID-19 illness. While a few recent studies reported in [9,41] have highlighted the application of unsupervised machine learning for the early detection and diagnosis of COVID-19, extracting valuable insights from the illness itself, it is worth noting that these studies did not extend this approach to wearable data in low-cost settings, as demonstrated in our work.

We conclude that there is a promising potential for using unsupervised learning techniques in detecting and monitoring COVID-19 using wearable devices’ data while taking care of many aspects, such as (a) the accuracy of the wearables, (b) the number of volunteers and the availability of the before and after COVID-19 infection data, and (c) using the best clustering algorithm that fits the data.

**Our Future Directions** would consider increasing the number of volunteers for a sounder conclusion. This step should encompass various populations, such as older adults, fully vaccinated, non-vaccinated, people with chronic cardiovascular diseases, and people from different geographical locations.

From an algorithmic point of view, it is worth investigating and exploring more clustering algorithms, such as Agglomerative Clustering, Mean Shift, and Affinity Propagation. The selection of algorithms should cover the different clustering techniques, namely centroid-based Clustering, density-based Clustering, distribution-based Clustering, and Hierarchical Clustering.

One notable clinical implication of our study is the development of an application that relies on clustering results and applies an algorithm to continuously monitor the physiological data acquired from wearables. This algorithm could detect if the results consistently fall within the abnormal cluster for a successive number of days, even before the onset of symptoms. Such an approach holds the potential to serve as an early warning system, enabling timely interventions and preventive measures for individuals at risk of COVID-19.

Finally, a more rigorous evaluation metrics of Clustering methods can be used, including Dunn’s Index, Rand index, Adjusted Rand Index, Calinski–Harabasz Index, and Mutual Information.

## Figures and Tables

**Table 1:** Selected HRV Features.

Feature Space	Feature	Description	Relationship with the COVID-19 Infection
**Time Domain**	RMSSD	The root mean square of the difference between successive RR intervals.	Studies (e.g., [8,40]) suggest that RMSSD exhibits a significantly lower tendency in COVID-19 patients.
	SDNN	The standard deviation of RR intervals in ms.	Hirten et al. [11] demonstrated a significant difference in SDNN between individuals with and without COVID-19 (*p* = 0.006).
**Frequency Domain**	LF/HF	The ratio between the low frequency and high frequency	Temple et al. [12] showed that the LF/HF increased significantly in positive COVID-19 patients.

**Table 2:** Clustering Algorithms.

Algorithm	Reasons	Essential Parameters
**K-means**	In addition to its simplicity, k-means showed high performance in various studies that addressed human biomedical data (e.g., [24,42]).	K is the number of clusters; we decided on k via the elbow method [43], as shown in the results of this work.
**DBSCAN (Density-based Spatial Clustering of Applications with Noise)**	DBSCAN can successfully identify noise in the data, ruling them out from clusters [44]	*ℇ* (eps) represents the maximum distance between two samples for a sample to be considered in the neighborhood of the other. We manually tested different values of *eps. MinPts*, which expresses the minimum number of neighbors within the eps range.
**Gaussian Mixture Model (GMM)**	This model was used to take the data variance into consideration when clustering. Moreover, this model could provide a probabilistic view of the data belonging to a given cluster.	*μ*: the mean of the Gaussian mixture components that define the center, a covariance matrix *Σ* that defines the distribution width, and a mixing probability *π* that identifies the size of the Gaussian function.

**Table 3:** Decision Tree Auto-Generated Rules From K-means Clustering Labels.

Class_Name	Instance_Count	Rule_List
**0**	4485	[0.98] (hr > 85.5) and (steps > 35.5)
**1**	42,001	[0.99] (hr > 85.5) and (steps <= 35.5)
**2**	57,394	[0.98] (hr <= 85.5)

**Table 4:** Evaluation of Supervised Classifiers Trained on K-means Clustering Labels.

Classifier	Accuracy	Precision	Recall
**Support Vector Machine (SVM) (Linear)**	0.884 ± 0.005	0.80 ± 0.112	0.817 ± 0.037
**Linear Discriminant Analysis**	0.850 ± 0.007	0.810 ± 0.020	0.754 ± 0.045
**Gaussian Naïve Bayes**	0.711 ± 0.005	0.863 ± 0.035	0.620 ± 0.087

**Table 5:** Decision Tree Auto-Generated Rules From K-means Clustering Labels of (HR and HRV).

Class_Name	Instance_Count	Rule_List
**0**	29,704	[0.97] (HR > 81.22) and (SDNN <= 0.07)
**1**	31,614	[0.98] (HR <= 81.22) and (SDNN <= 0.07)
**2**	15,316	[0.88] (HR <= 81.22) and (SDNN > 0.07) [0.84] (HR > 81.22) and (SDNN > 0.07)

## Data Availability

Will be made public soon.
